# Facial Eczema*Abstract of a Clinical Lecture delivered at the Hospital College of Medicine.

**Published:** 1897-07

**Authors:** John E. Hays

**Affiliations:** Professor of Dermatology, Etc., in the Hospital College of Medicine, Louisville, Kentucky


### FACIAL ECZEMA.*

BY JOHN E. HAYS, M. D.

Professor of Dermatology, Etc., in the Hospital College of Medicine, Louisville, Kentucky.

This patient, a female, aged two years, is brought to us for treatment to-day for the first time. The history given by the mother is that an eruption appeared on the child's face two months ago, which has existed continuously since that time. She also noticed that the child had some lung affection for the last two weeks. You notice an eczema, involving both cheeks; although the disease has existed, according to the history, for two months, it seems to have been confined to this locality. In this variety, which is a squamous eczema, if you pinch up the skin between your fingers you will find it very mnch thickened, inflamed, and hard.

We have exhibited before you during the session a great many cases of eczema. It is a chronic inflammatory trouble confined to the superficial layers of the skin. In the case before us we see no evidence of moisture at the present time, although I suppose at some time the patches have been moist. For instance, after washing the child's face, we could probably see oozing from these little vessels the liquor sanguinis, which usually stands out in small beadlets. It is always made worse by washing. The disease in this case does not seem to have shown any tendency to spread; in many instances an eczema existing for two months will have spread over the entire face and even the scalp.

The mother tell us that the disease seems to be very much aggravated when she washes the child's face. Often in cases of eczema quite as much depends upon what we proscribe as what we prescribe in the way of treatment, and it is my custom to tell the mother not to wash the face of the child until the disease has been thoroughly cured. If it becomes necessary at any time to use some cleansing agent, I advise the application of some pure sweet oil. Saturate a piece of soft cloth or absorbent cotton with sweet oil, and pass it over the patch several times, and in this way you can cleanse it as perfectly as could be done with water, and cleansing in this way does

* Abstract of a Clinical Lecture delivered at the Hospital College of Medicine.

not interfere with the process of healing. You will find it almost impossible to cure a patch of eczema of the face if the mother or nurse is allowed to wash it, particularly if some form of cheap, irritating soap is used.

The proper treatment in this case is to employ some mild protective salve, which contains also a remedy capable of destroying the parasite which is probably the cause of the trouble. We will prescribe the ammoniate of mercury, oxide of zinc, powdered starch, and vaselin, as follows:

| | | |
|---|---|---|
| ℞ | Ammoniate of mercury................. | 15 grains, |
| | Oxide of zinc........................... | 1 dram, |
| | Powdered starch........................ | 1 dram, |
| | Vaselin.................................... | 6 drams. |

Mix, and instruct the mother to keep the patches constantly covered with this salve, using a small amount several times during the day. The child's face should not be washed with water until the disease is cured, but if cleansing becomes necessary, it should be done with sweet oil, as we have described. —*Pediatrics.*

